# Supplementary material for: The Mediator co-activator complex regulates Ty1 retromobility by controlling the balance between Ty1i and Ty1 promoters
Source: PLoS Genet. 2018 Feb 20;14(2):e1007232. doi: 10.1371/journal.pgen.1007232 (PMC5834202; doi:10.1371/journal.pgen.1007232)
Supplement: S1 Fig — (A) Western blot of total cell lysates from wild-type, spt3Δ, and Mediator subunit deletion strains probed for Gag using an anti-VLP antibody. (B) Quantitation of p49/45 levels relative to α-actin from (A), normalized to WT. (PPTX) [file pgen.1007232.s001.pptx]

## Slide 1
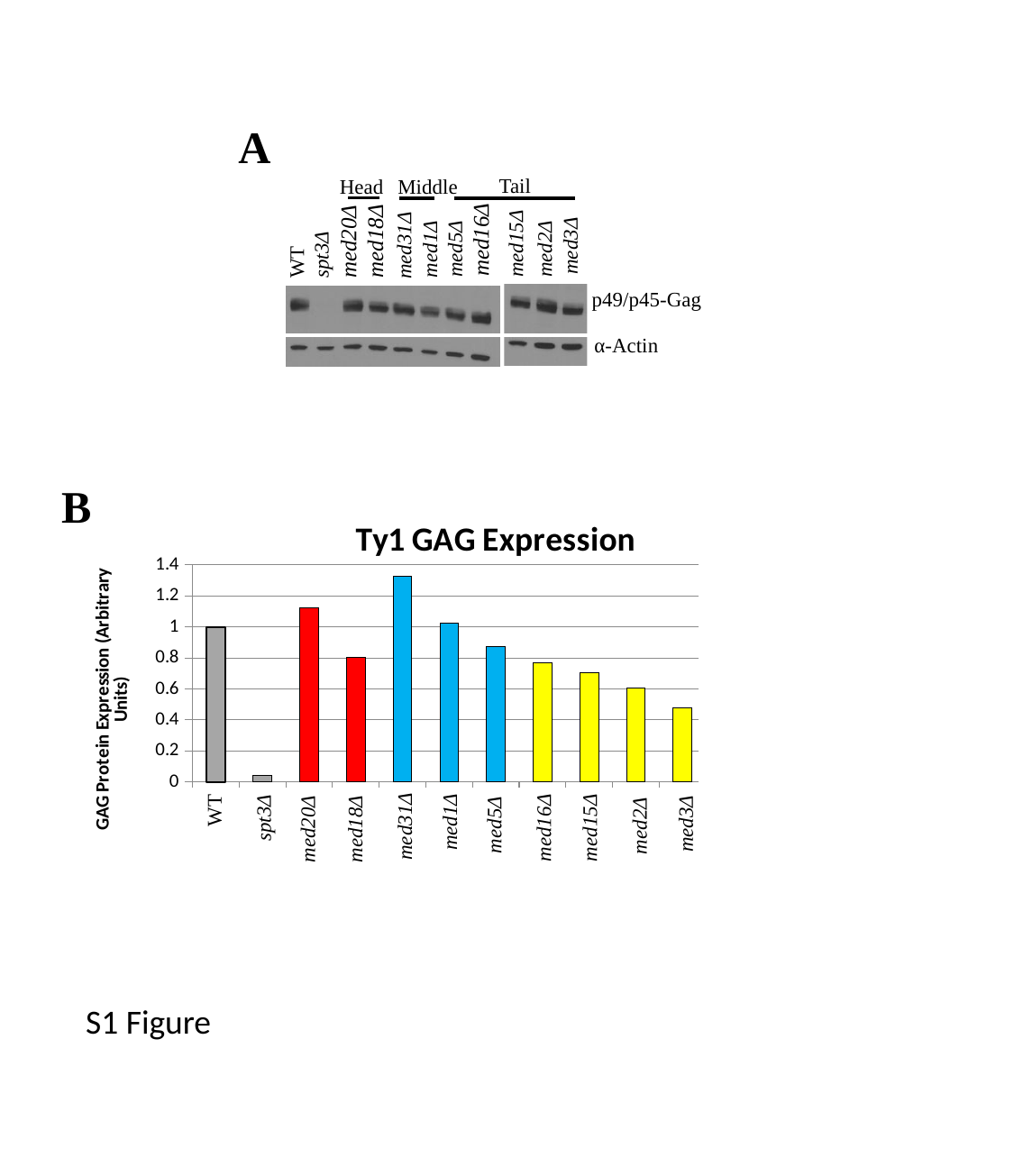

A
Tail
Middle
Head
med16Δ
med3Δ
med18Δ
med20Δ
med31Δ
med15Δ
med2Δ
med5Δ
med1Δ
spt3Δ
WT
p49/p45-Gag
α-Actin
B
### Chart: Ty1 GAG Expression
| Category | |
|---|---|
| WT | 1.0 |
| spt3Δ | 0.04412181259725 |
| srb2Δ | 1.12554578410391 |
| srb5Δ | 0.803436310246293 |
| soh1Δ | 1.325390811561414 |
| med1Δ | 1.02654863174855 |
| nut1Δ | 0.875515010976514 |
| sin4Δ | 0.770990613888827 |
| gal11Δ | 0.704551234000201 |
| med2Δ | 0.605788121624043 |
| pgd1Δ | 0.477343940494772 |
| cdk8Δ | 0.783624467885877 |
| med13Δ | 1.124894768375715 |
| srb11Δ | 0.809109513119064 |
| srb8Δ | 0.786344669205906 |WT
med1Δ
spt3Δ
med3Δ
med5Δ
med2Δ
med31Δ
med15Δ
med20Δ
med18Δ
med16Δ
S1 Figure
