# Supplementary figures and images for: The Mediator co-activator complex regulates Ty1 retromobility by controlling the balance between Ty1i and Ty1 promoters
Source: PLoS Genet. 2018 Feb 20;14(2):e1007232. doi: 10.1371/journal.pgen.1007232 (PMC5834202; doi:10.1371/journal.pgen.1007232)

## Slide 1
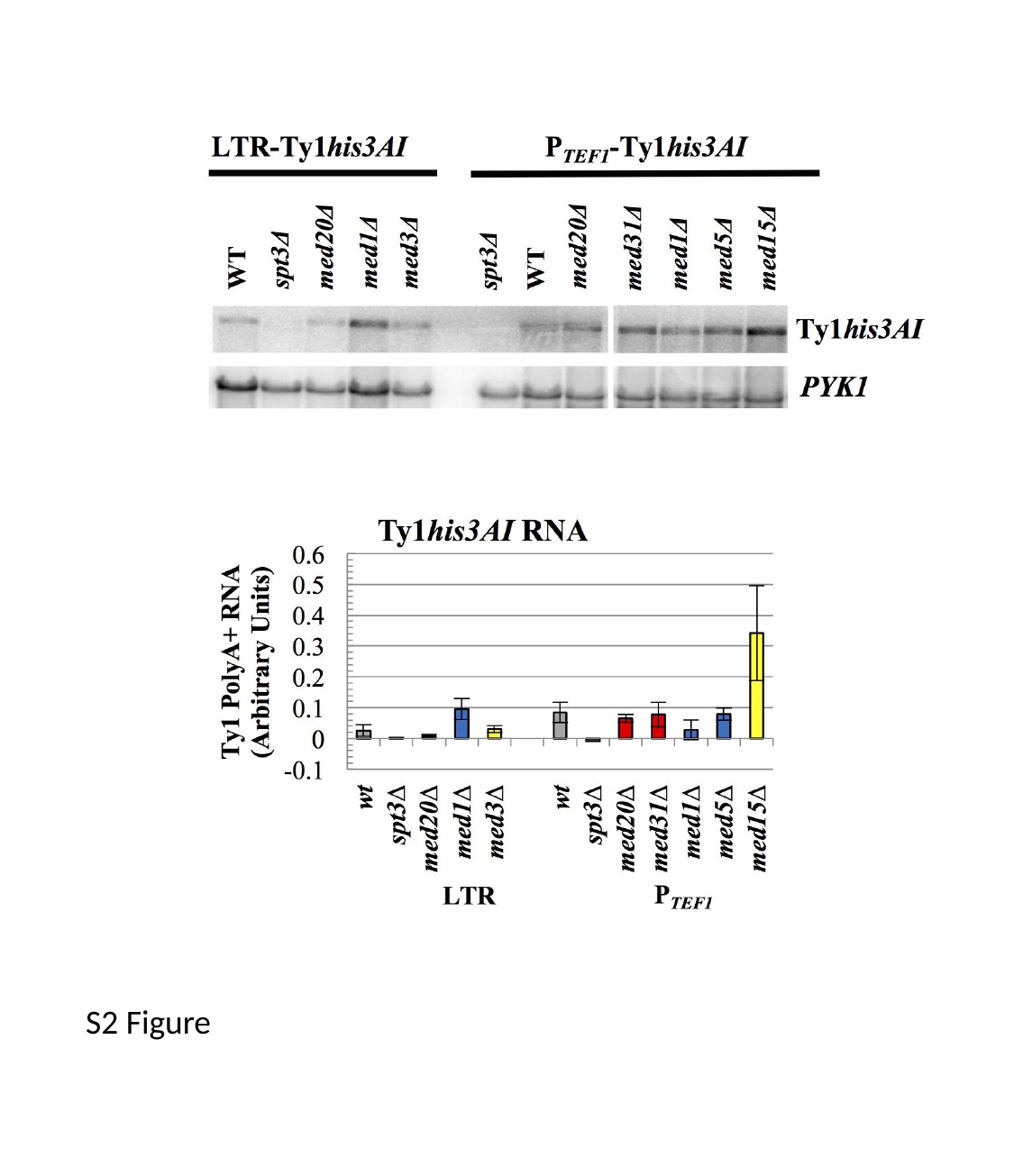

S2 Figure

Supplement: S2 Fig — The blot was probed with a sense-strand HIS3 riboprobe to detect Ty1his3AI and Ty1ihis3AI RNA. All lanes shown are from a single gel. Note the absence of any band below the Ty1his3AI transcript (compare Fig 5A). The values reported in the graph are the average ratio of Ty1his3AI RNA relative to PYK1 RNA in two biological replicates. Bars are color-coded as in Fig 2A: gray, WT and spt3Δ; red, Mediator head module deletion; blue, middle module deletion; yellow, tail module deletion. All error bars represent range of measurement. (PPTX) [file pgen.1007232.s002.pptx]

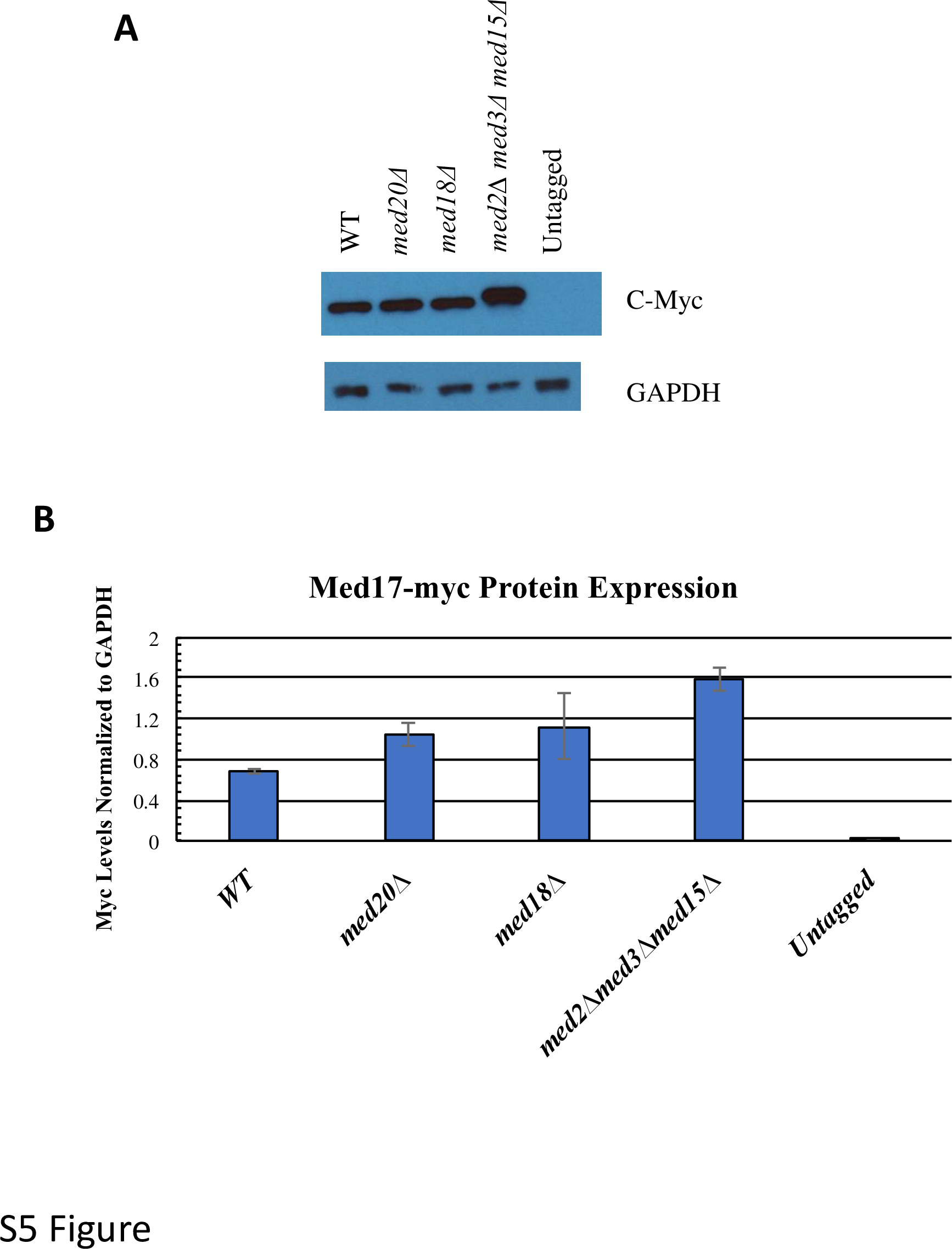

Supplement: S5 Fig — (A) Western blot of total cell lysates from wild-type and mutant yeast expressing c-Myc tagged Med17 (first four lanes) and from an untagged control strain (last lane), probed using an antibody against c-Myc and against GAPDH. (B) Quantitation of two biological replicate experiments as in (A). Error bars indicate range of measurement. (TIF) [file pgen.1007232.s005.tif]
