# Supplementary material for: The Mediator co-activator complex regulates Ty1 retromobility by controlling the balance between Ty1i and Ty1 promoters
Source: PLoS Genet. 2018 Feb 20;14(2):e1007232. doi: 10.1371/journal.pgen.1007232 (PMC5834202; doi:10.1371/journal.pgen.1007232)
Supplement: S3 Fig — Top, untagged control subjected to ChIP-seq using anti-myc antibody. Bottom, occupancy of myc-tagged Med15 and Med17 over averaged Ty1 elements in med18Δ kin28-AA yeast. Ty1 elements begin at 0 kb on the x-axis, and the Ty1 TSS at +238 and Ty1i TSS at +1000 are marked by green bars on the x-axis. Note the absence of any Mediator peak at the Ty1i TSS. (PPTX) [file pgen.1007232.s003.pptx]

## Slide 1
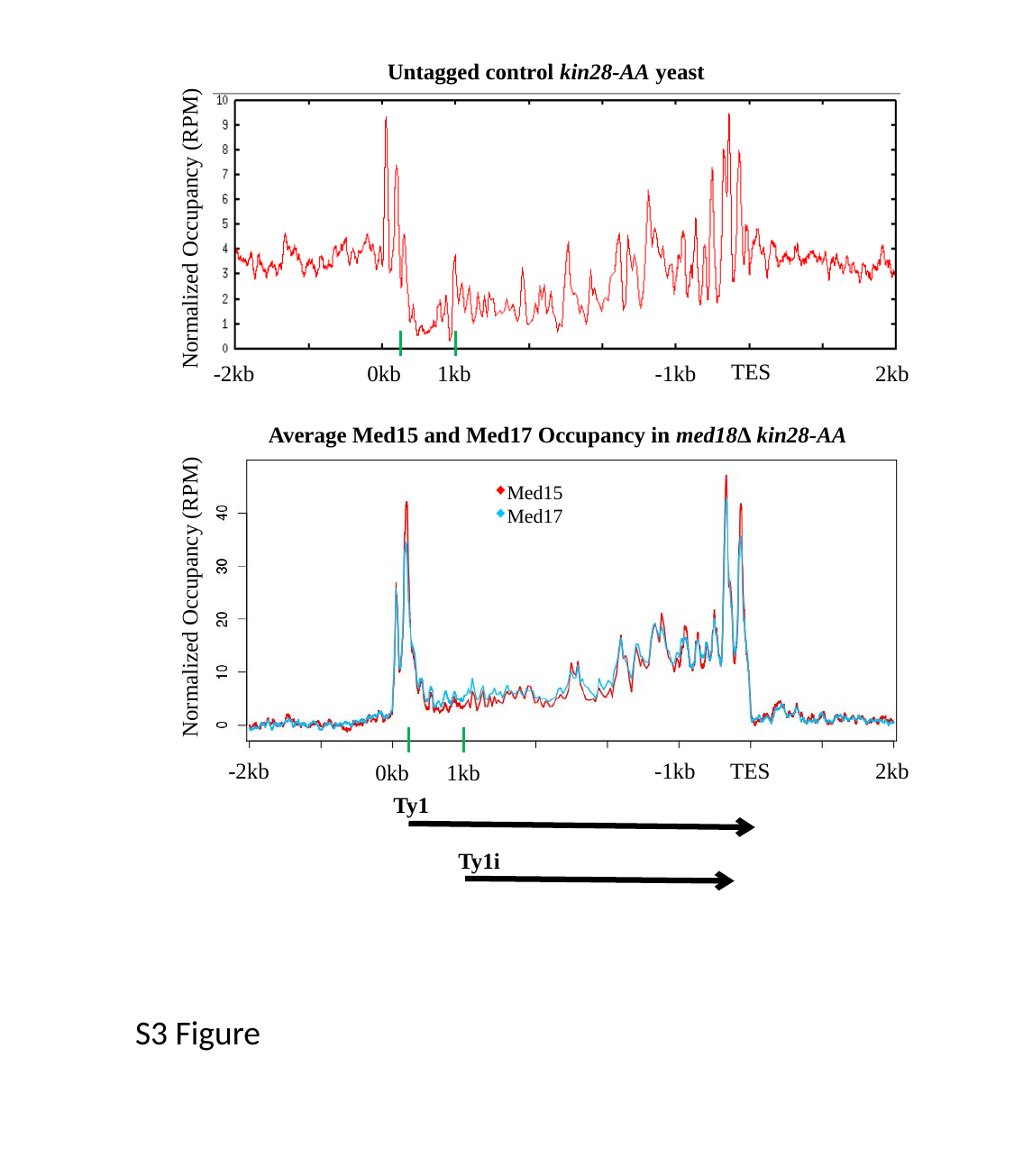

Untagged control kin28-AA yeast
Normalized Occupancy (RPM)
TES
 0kb
 1kb
-1kb
 2kb
-2kb
Average Med15 and Med17 Occupancy in med18∆ kin28-AA yeast
Med15
Med17
Normalized Occupancy (RPM)
-1kb
TES
 2kb
-2kb
 1kb
 0kb
Ty1
Ty1i
S3 Figure
