# Supplementary material for: The Mediator co-activator complex regulates Ty1 retromobility by controlling the balance between Ty1i and Ty1 promoters
Source: PLoS Genet. 2018 Feb 20;14(2):e1007232. doi: 10.1371/journal.pgen.1007232 (PMC5834202; doi:10.1371/journal.pgen.1007232)
Supplement: S4 Fig — Data is the same as in Figs 5 and 6. (PPTX) [file pgen.1007232.s004.pptx]

## Slide 1
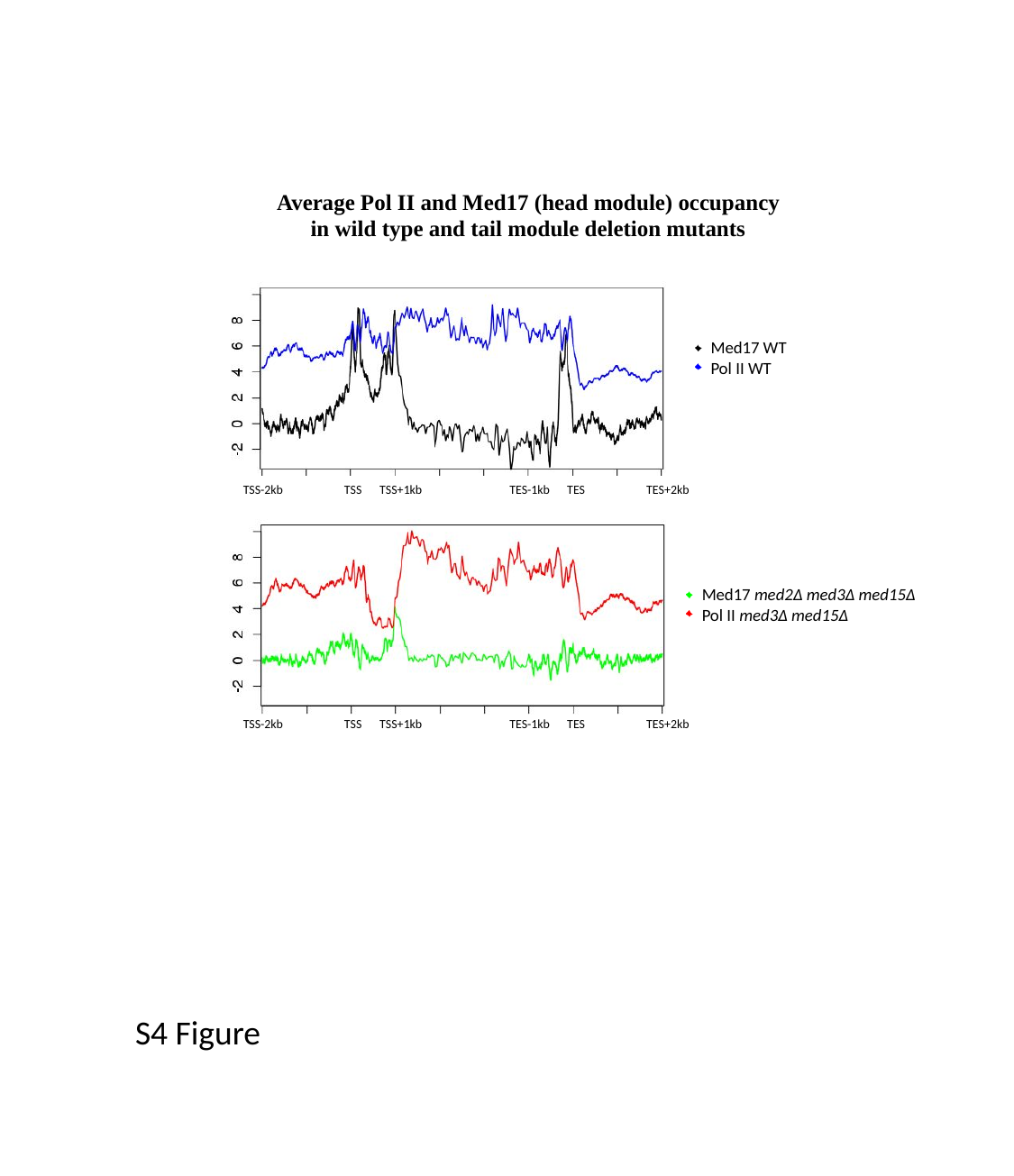

Average Pol II and Med17 (head module) occupancy in wild type and tail module deletion mutants
Med17 WT
Pol II WT
TSS-2kb TSS TSS+1kb TES-1kb TES TES+2kb
Med17 med2∆ med3∆ med15∆
Pol II med3∆ med15∆
TSS-2kb TSS TSS+1kb TES-1kb TES TES+2kb
S4 Figure
