# Supplementary material for: The Mediator co-activator complex regulates Ty1 retromobility by controlling the balance between Ty1i and Ty1 promoters
Source: PLoS Genet. 2018 Feb 20;14(2):e1007232. doi: 10.1371/journal.pgen.1007232 (PMC5834202; doi:10.1371/journal.pgen.1007232)
Supplement: S6 Fig — Top: Schematic of the GAL1:Ty1ΔPOL cassette in pGTy1ΔPOL showing forward primer locations for detection of Ty1 RNA (blue) versus Ty1i RNA (red). Note that because Ty1i is contained within Ty1, the Ty1i primer reports both Ty1i and Ty1 transcripts. Both amplifications utilized the same reverse primer (purple), that crosses the deletion junction and contains sequences unique to the pGTy1ΔPOL element. A reverse primer specific for ACT1 was also used to synthesize cDNA used as a template for the PCR amplification. No PCR product was detected using the ΔPOL reverse primer when RNA from yeast lacking pGTy1ΔPOL was used as template. Bottom: Reverse Transciption-PCR reactions using polyA+ RNA isolated from strains of the indicated genotype bearing plasmid pGTy1ΔPOL, and grown in glucose-containing broth. Aliquots were taken from reactions at the indicated number of cycles and analyzed by agarose gel electrophoresis. RT-PCR amplification products using Ty1, Ty1i, and ACT1 primers are indicated. We do not know the origin of the apparently spurious, lower molecular weight bands observed. The same WT samples were used for all panels; results were similar for a second biological replicate of all three samples (WT, med20Δ, and med3Δ). (PPTX) [file pgen.1007232.s006.pptx]

## Slide 1
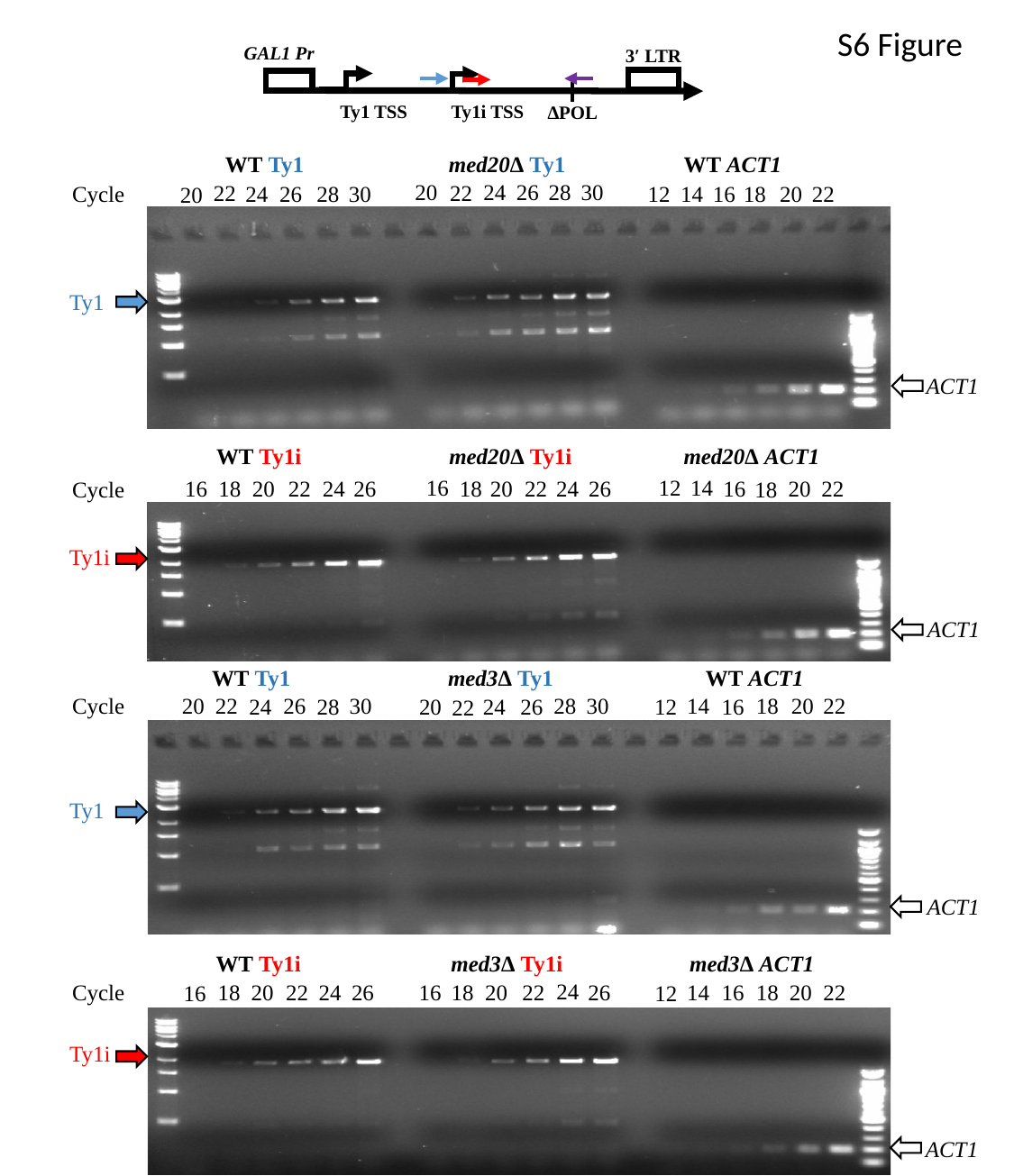

S6 Figure
GAL1 Pr
3′ LTR
Ty1i TSS
Ty1 TSS
∆POL
WT ACT1
med20∆ Ty1
WT Ty1
20
26
28
30
24
22
22
20
24
12
14
Cycle
16
26
22
18
30
28
20
Ty1
ACT1
med20∆ ACT1
med20∆ Ty1i
WT Ty1i
14
12
16
22
22
18
18
24
26
22
16
20
24
26
20
20
16
Cycle
18
Ty1i
ACT1
med3∆ Ty1
WT Ty1
WT ACT1
26
18
20
22
14
22
30
28
30
Cycle
20
28
16
26
20
12
24
24
22
Ty1
ACT1
WT Ty1i
med3∆ Ty1i
med3∆ ACT1
24
24
26
14
20
Cycle
18
22
16
18
20
22
26
16
18
20
22
12
16
Ty1i
ACT1
