# Supplementary material for: The Mediator co-activator complex regulates Ty1 retromobility by controlling the balance between Ty1i and Ty1 promoters
Source: PLoS Genet. 2018 Feb 20;14(2):e1007232. doi: 10.1371/journal.pgen.1007232 (PMC5834202; doi:10.1371/journal.pgen.1007232)
Supplement: S1 Table — (DOCX) [file pgen.1007232.s007.docx]

**S1 Table: Yeast strains used in this work**

| Strain | Genotype | Source |
| --- | --- | --- |
| BY4741 | *MATa, his3∆1, leu2∆0, met15∆0, lys2∆0, ura3∆0* | [51] |
| BY4741 spt3∆ | *spt3∆::kanMX* derivative of BY4741 | Open Biosystems |
| BY4741 med20∆ | *med20∆::kanMX* derivative of BY4741 | Open Biosystems |
| BY4741 med18∆ | *med18∆::kanMX* derivative of BY4741 | Open Biosystems |
| BY4741 med31∆ | *med31∆::kanMX* derivative of BY4741 | Open Biosystems |
| BY4741 med1∆ | *med1∆::kanMX* derivative of BY4741 | Open Biosystems |
| BY4741 med5∆ | *med5∆::kanMX* derivative of BY4741 | Open Biosystems |
| BY4741 med3∆ | *med3∆::kanMX* derivative of BY4741 | Open Biosystems |
| BY4741 cdk8∆ | *cdk8∆::kanMX* derivative of BY4741 | Open Biosystems |
| BY4741 med13∆ | *med13∆::kanMX* derivative of BY4741 | Open Biosystems |
| BY4741 cycc∆ | *cycc∆::kanMX* derivative of BY4741 | Open Biosystems |
| BY4741 med12∆ | *med12∆::kanMX* derivative of BY4741 | Open Biosystems |
| BY4741 med15∆ | *med15∆::ura3* derivative of BY4741 | [69] |
| JC3212 | *MATa, his3∆1, leu2∆0, met15∆0, ura3∆0,* Ty1*his3AI-*3114 | [50] |
| JC3212 spt3∆ | *spt3∆::kanMX* derivative of JC3212 | [15] |
| JC3212 xrn1∆ | *xrn1∆::kanMX* derivative of JC3212 | [15] |
| JC3212 med20∆ | *med20∆::kanMX* derivative of JC3212 | This study |
| JC3212 med18∆ | *med18∆::kanMX* derivative of JC3212 | This study |
| JC3212 med31∆ | *med31∆::kanMX* derivative of JC3212 | This study |
| JC3212 med1∆ | *med1∆::kanMX* derivative of JC3212 | This study |
| JC3212 med5∆ | *med5∆::kanMX* derivative of JC3212 | This study |
| JC3212 med16∆ | *med16∆::kanMX* derivative of JC3212 | This study |
| JC3212 med2∆ | *med2∆::kanMX* derivative of JC3212 | This study |
| JC3212 med3∆ | *med3∆::kanMX* derivative of JC3212 | This study |
| JC3212 med15∆ | *med15∆::URA3* derivative of JC3212 | This study |
| JC6464 | *MATa, his3∆1, leu2∆0, met15∆0, ura3∆0,* Ty1*kanMXAI-*6464 | This study |
| JC6464  med15∆ | *med15∆::URA3* derivative of JC6464 |  |
| LTRTy1 | Derivative of BY4741 containing plasmid pBJC567 | This study |
| LTRTy1 spt3∆ | *spt3∆::kanMX* derivative of LTRTy1 | This study |
| LTRTy1 med20∆ | *med20∆::kanMX* derivative of LTRTy1 | This study |
| LTRTy1 med18∆ | *med18∆::kanMX* derivative of LTRTy1 | This study |
| LTRTy1 med31∆ | *med31∆::kanMX* derivative of LTRTy1 | This study |
| LTRTy1 med1∆ | *med1∆::kanMX* derivative of LTRTy1 | This study |
| LTRTy1 med5∆ | *med5∆::kanMX* derivative of LTRTy1 | This study |
| LTRTy1 med3∆ | *med3∆::kanMX* derivative of LTRTy1 | This study |
| P*_TEF1_* | Derivative of BY4741 containing plasmid pBJC1250 | This study |
| P*_TEF1_* spt3∆ | *spt3∆::kanMX* derivative of P*_TEF1_* | This study |
| P*_TEF1_* med20∆ | *med20∆::kanMX* derivative of P*_TEF1_* | This study |
| P*_TEF1_* med18∆ | *med18∆::kanMX* derivative of P*_TEF1_* | This study |
| P*_TEF1_* med31∆ | *med31∆::kanMX* derivative of P*_TEF1_* | This study |
| P*_TEF1_* med1∆ | *med1∆::kanMX* derivative of P*_TEF1_* | This study |
| P*_TEF1_* med5∆ | *med5∆::kanMX* derivative of P*_TEF1_* | This study |
| P*_TEF1_* med15∆ | *med15∆::URA3* derivative of P*_TEF1_* | This study |
| P*_TEF1_* med3∆ | *med3∆::kanMX* derivative of P*_TEF1_* | This study |
| pGTy1ΔPOL | Derivative of JC3212 containing plasmid pBJC80 | This study |
| pGTy1ΔPOL spt3∆ | *spt3∆::kanMX* derivative of pGTy1∆POL | This study |
| pGTy1ΔPOL med20∆ | *med20∆::kanMX* derivative of pGTy1∆POL | This study |
| pGTy1ΔPOL med31∆ | *med31∆::kanMX* derivative of pGTy1∆POL | This study |
| pGTy1ΔPOL med1∆ | *med1∆::kanMX* derivative of pGTy1∆POL | This study |
| pGTy1ΔPOL med5∆ | *med5∆::kanMX* derivative of pGTy1∆POL | This study |
| pGTy1ΔPOL med2∆ | *med2∆::kanMX* derivative of pGTy1∆POL | This study |
| pGTy1ΔPOL med3∆ | *med3∆::kanMX* derivative of pGTy1∆POL | This study |
| TBY128 | *MATα, ade2-1, his3-11,15, leu2-3,112, trp1-1, ura3-1, can1-100 tor1-1, fpr1::NAT, RPL13A-2xFKB12::TRP1, KIN28-FRB::HIS3 MED15-18MYC::klURA3* | This study |
| TBY129 | *MATα, ade2-1, his3-11,15, leu2-3,112, trp1-1, ura3-1, can1-100 tor1-1, fpr1::NAT, RPL13A-2xFKB12::TRP1, KIN28-FRB::HIS3 MED17-9MYC::klTRP1* | This study |
| TBY130 | *MATα, ade2-1, his3-11,15, leu2-3,112, trp1-1, ura3-1, can1-100 tor1-1, fpr1::NAT, RPL13A-2xFKB12::TRP1, KIN28-FRB::HIS3 MED15-18MYC::klURA3 med20::KanMX* | This study |
| TBY131 | *MATα, ade2-1, his3-11,15, leu2-3,112, trp1-1, ura3-1, can1-100 tor1-1, fpr1::NAT, RPL13A-2xFKB12::TRP1, KIN28-FRB::HIS3 MED17-9MYC::klTRP1 med20::KanMX* | This study |
| TBY132 | *MATα, ade2-1, his3-11,15, leu2-3,112, trp1-1, ura3-1, can1-100 tor1-1, fpr1::NAT, RPL13A-2xFKB12::TRP1, KIN28-FRB::HIS3 MED15-18MYC::klURA3 med18::KanMX* | This study |
| TBY133 | *MATα, ade2-1, his3-11,15, leu2-3,112, trp1-1, ura3-1, can1-100 tor1-1, fpr1::NAT, RPL13A-2xFKB12::TRP1, KIN28-FRB::HIS3 MED17-9MYC::klTRP1 med18::KanMX* | This study |
| RMY 1010-A5D | *MATa med3::KanMX med15::KanMX med2::KanMX leu2∆0 met15∆0 ura3∆0 trp1- tor1-1 fpr1::NAT RPL13A-2XFKBP::TRP1 KIN28-FRB::hph MED17-18MYC::klURA3* | This study |
